# Statin Use and Cognitive Function: Population-Based Observational Study with Long-Term Follow-Up

**DOI:** 10.1371/journal.pone.0115755

**Published:** 2014-12-26

**Authors:** Hanneke Joosten, Sipke T. Visser, Marlise E. van Eersel, Ron T. Gansevoort, Henk J. G. Bilo, Joris P. Slaets, Gerbrand J. Izaks

**Affiliations:** 1 University of Groningen, University Medical Center Groningen (UMCG), Department of Internal medicine, Groningen, The Netherlands; 2 University of Groningen, Department of Pharmacy, PharmacoEpidemiology and PharmacoEconomics (PE2), Groningen, The Netherlands; 3 University of Groningen, University Medical Center Groningen (UMCG), University Center for Geriatric Medicine, Groningen, The Netherlands; 4 University of Groningen, University Medical Center Groningen (UMCG), Department of Internal medicine, Division of Nephrology, Groningen, The Netherlands; 5 Department of Internal medicine, Diabetes Centre, Isala Clinics, Zwolle, The Netherlands; University of Granada, Spain

## Abstract

We aimed to evaluate the association between statin use and cognitive function. Cognitive function was measured with the Ruff Figural Fluency Test (RFFT; worst score, 0; best score, 175 points) and the Visual Association Test (VAT; low performance, 0–10; high performance, 11–12 points) in an observational study that included 4,095 community-dwelling participants aged 35–82 years. Data on statin use were obtained from a computerized pharmacy database. Analysis were done for the total cohort and subsamples matched on cardiovascular risk (N = 1232) or propensity score for statin use (N = 3609). We found that a total of 904 participants (10%) used a statin. Statin users were older than non-users: mean age (SD) 61 (10) vs. 52 (11) years (*p*<0.001). The median duration of statin use was 3.8 (interquartile range, 1.6–4.5) years. Unadjusted, statin users had worse cognitive performance than non-users. The mean RFFT score (SD) in statin users and non-users was 58 (23) and 72 (26) points, respectively (*p*<0.001). VAT performance was high in 261 (29%) statin users and 1351 (43%) non-users (*p*<0.001). However, multiple regression analysis did not show a significant association of RFFT score with statin use (B, −0.82; 95%CI, −2.77 to 1.14; *p* = 0.41) nor with statin solubility, statin dose or duration of statin use. Statin users with high doses or long-term use had similar cognitive performance as non-users. This was found in persons with low as well as high cardiovascular risk, and in younger as well as older subjects. Also, the mean RFFT score per quintile of propensity score for statin use was comparable for statin users and non-users. Similar results were found for the VAT score as outcome measure. In conclusion, statin use was not associated with cognitive function. This was independent of statin dose or duration of statin use.

## Introduction

Cardiovascular risk factors are not only associated with coronary heart disease and stroke but also with cognitive dysfunction, due to shared atherosclerotic complications [1]–[3]. As dyslipidemia is a major cardiovascular risk factor, it is not surprising that dyslipidemia in midlife associates with cognitive dysfunction and dementia in later life [4]–[6]. Dyslipidemia can be effectively improved by statins. Therefore, it can be hypothesized that statin use has beneficial effects on cognitive function.

Up till now, two randomized controlled trials (RCTs) have studied the effect of statin use on cognitive function and both failed to show a beneficial effect [7], [8]. One explanation could be the relatively short duration of these RCTs. Possibly, statins need a longer period to have a positive effect on cognitive function. Notably, several observational studies that had a longer duration of follow-up than these RCTs suggested a favorable effect [9]–[16]. For example, elderly with>4 years of continuous statin use had less cognitive decline than subjects who used statins less intensively [12]. In line with these data a recent meta-analysis found no short-term effects on cognitive function, whereas long-term use might be associated with a beneficial role in the prevention of dementia [17]. Duration of statin use might thus influence results [18], and this should formally be tested. Due to the financial burden, RCTs of longer duration probably are not feasible. Therefore, long-term observational studies are necessary to evaluate whether long-term statin use prevents cognitive decline [19]. However, confounding by indication can be an important limitation of observational studies as, generally, statin users have a different cardiovascular risk profile than non-users, and this type of bias needs to be addressed.

The aim of this observational study was to evaluate the association between statin use and cognitive function in a large community-based population aged 35–82 years with>10 year follow-up data on statin use, and to study whether duration of treatment influences this association. All participants underwent a detailed assessment of cardiovascular risk factors that was used to adjust for confounding by indication.

## Methods

### Study population

This study was part of the third survey of the Prevention of REnal and Vascular ENd-stage Disease (PREVEND) cohort study. The PREVEND study is a prospective cohort study investigating the natural course of increased albuminuria and its association with renal and cardiovascular disease [20], [21]. In brief, at baseline, 8,592 participants aged 28–75 years were selected from inhabitants of the city of Groningen (The Netherlands) based on their urinary albumin excretion (UAE): 2,592 with UAE<10 mg/L and 6,000 with UAE≥10 mg/L. These participants completed the baseline survey in 1997–1998 and were followed over time. A total of 6,894 participants (80%) completed the second survey in 2001–2003, and 5,862 (68%) the third survey in 2003–2006. All surveys included assessment of demographic characteristics, cardiovascular risk factors and haematological and biochemical parameters. In the third survey, measurement of cognitive function was added to the study protocol.

### Standard protocol approvals and participant consents

All participants gave written informed consent. The PREVEND study was approved by the medical ethics committee (METc) of University Medical Center Groningen and conducted in accordance with the guidelines of the Helsinki declaration.

### Cognitive function

The RFFT was the primary outcome measure for cognitive function [22]–[24]. In brief, the RFFT requires participants to draw as many unique designs as possible within a set time limit by connecting dots in a different pattern while avoiding repetitions of designs. The RFFT is generally seen as a measure of executive function [22], [23], [25]. The main outcome measure of the RFFT is the total number of unique designs. The lowest (worst) RFFT score is 0 points, the highest (best) score is 175 points [22], [23]. Each RFFT was scored by two trained and independent examiners.

The VAT was used as a secondary outcome measure for cognitive function. The VAT is a brief learning task that is designed to detect impaired memory, including anterograde amnesia [26]. The test consists of six drawings of pairs of interacting objects. The participant needs to name each object and, later, is presented with one object from the pair and asked to name the other. The lowest (worst) score is 0 points and the highest (best) score is 12 points [26].

### Statin use

Subject-specific information on statin use was obtained from IADB.nl, a database comprising pharmacy-dispensing data from all community pharmacies in the city of Groningen, The Netherlands, since 1999 [27]. These data include information on the name of the drug dispensed, Anatomical Therapeutical Chemical (ATC) classification, solubility (hydrophilic or lipophilic), date of prescription, number of days the drug was prescribed and the number of prescribed defined daily doses (DDD). DDD is defined by the WHO as the drug units representing dosages with approximately similar efficacy. One DDD corresponds to the following dosage for each statin respectively: Simvastatin 30 mg, Pravastatin 30 mg, Fluvastatin 60 mg, Atorvastatin 20 mg and Rosuvastatin 10 mg.

Statin use was defined as at least one prescription in the period preceding the third survey of the PREVEND study with at least one prescription ≤100 days before performance of the cognitive function tests. Statin use was not only defined dichotomously (yes/no), but also explored by statin solubility, daily dose (defined as the mean amount of DDDs per day), duration of use (years) and cumulative dose over time (overall amount of DDDs used).

### Other variables

Educational level was classified by highest achieved degree based on a questionnaire and divided into four groups: Primary school level, 0–8 years of education; lower secondary level, 9–12 years; higher secondary level, 13–15 years; university level, ≥16 years. A history of cardiovascular events was defined as a prior cardiac, cerebrovascular or peripheral vascular event requiring hospitalization. Past medical history was derived from a questionnaire at baseline and the Dutch national registry of hospital discharge diagnoses during follow-up. Diabetes mellitus (DM) was defined as a fasting glucose ≥7 mmol/L, a non-fasting glucose ≥11.1 mmol/L or the use of glucose-lowering drugs. Hypertension was defined as use of antihypertensive drugs or systolic blood pressure>140 mmHg or diastolic blood pressure>90 mmHg. Albuminuria was calculated from the mean of two 24-h urinary collections and elevated albuminuria was defined as ≥30 mg/24 h.

### Matching

**Figure 1:**
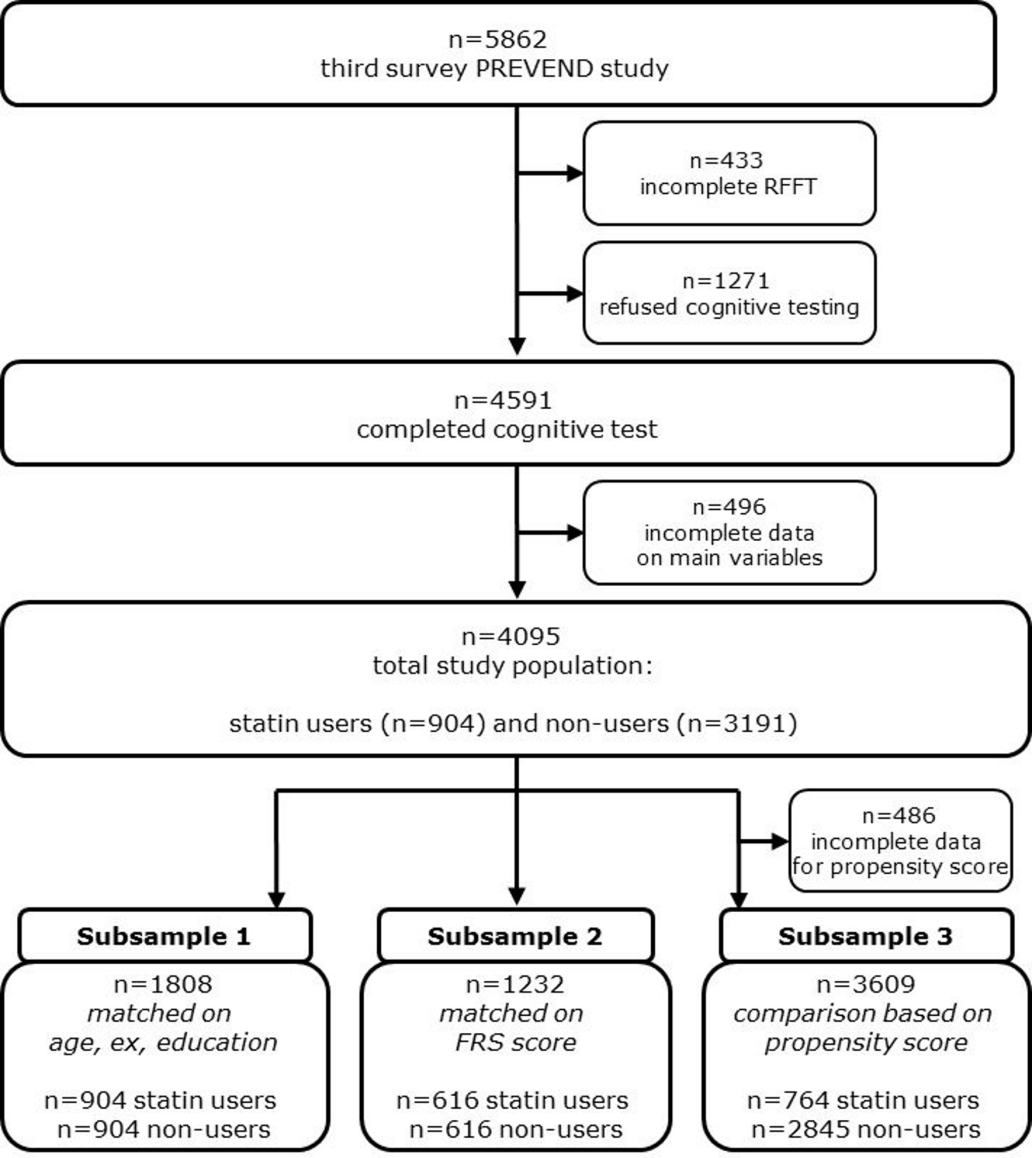
Flow diagram of the selection of the study population.

For the specific aim of this study, we created three additional (partly overlapping) subsamples from the total study population (*Fig. 1*): 1. a subsample of statin users and non-users who were matched one-to-one on age, sex and educational level; 2. a subsample of statin users and non-users who were matched one-to-one on cardiovascular risk profile; 3. a subsample of statin users and non-users who were compared by quintile of propensity score for statin use.

### Cardiovascular risk

Overall cardiovascular risk was measured by the Framingham risk score for general cardiovascular disease (FRS) [28], a composite measure designed to predict the risk of developing a cardiovascular event within the next ten years. Calculation of the FRS is based on age, sex, DM, current smoker status, SBP, total cholesterol, HDL-cholesterol and use of blood pressure lowering agents. A higher FRS is associated with a higher risk of a new cardiovascular event: the lowest score is −5 (risk <1%) and the highest score 33 (risk>30%) [28].

### Propensity score

A propensity score balances covariates in observational studies associated with the prescription of drugs and is used to reduce bias by indication in non-randomized studies. In this study, the propensity score for statin therapy was calculated by a logistic regression model including age, sex, educational level, history of CVD, smoking, DM, hypertension, BMI and albuminuria [29].

### Statistical analysis

Statistical analysis was performed with IBM SPSS Statistics version 20 (IBM Corporation, Amonk, NY). Normally distributed data are presented as mean and standard deviation (SD) and skewed data as median and interquartile range (IQR). Differences between two samples were tested by *t*-test or, if appropriate, Mann-Whitney *U* test. Differences between more than 2 samples were tested by ANOVA. Adjusted RFFT scores were calculated by analysis of covariance (ANCOVA). Adjustment was made for age, sex, educational level, history of CVD, DM, hypertension, BMI, smoking, alcohol use, lipid levels, family history of CVD and propensity score.

#### Multiple linear regression models

Multiple linear regression analysis was used to model the independent association of RFFT score with statin use. In all regression models, RFFT score was the dependent variable, and a measure of statin use was the independent variable (statin use (yes/no), statin solubility, cumulative statin dose, duration of statin use, or mean daily dose). The regression models were adjusted for three different sets of possible confounders: model 1, adjusted for age, sex and educational level; model 2, adjusted for the covariates of model 1 plus DM, hypertension, history of CVD, smoking, BMI, albuminuria and lipid levels. To avoid overcorrection the propensity score was not included as a covariate in any of the regression models on top of the single covariates (which were also used to calculate the propensity score).

Similar analyses were performed for VAT score as the cognitive outcome measure. Because of its skewed distribution, the VAT score was dichotomized at the median into low performance (≤10 points) and high performance (≥11 points). Accordingly, the association of VAT performance with statin use was evaluated by logistic regression analysis (see above).

Because the PREVEND cohort is enriched for subjects with higher levels of albuminuria which may be negatively associated with cognitive function [30], analyses were repeated in a subsample of the PREVEND cohort which is representative for the general population (prevalence of elevated albuminuria 8%).

## Results

All 5,862 participants of the third survey were reviewed. Subjects with no or incomplete data on cognitive function (n = 1704), statin use or other main variables (n = 63) were excluded (*Fig. 1*). The final overall study population thus comprised 4095 subjects.

The three (partly overlapping) subsamples from the final study population (*Fig. 1*) consisted of 1808 statin users and non-users matched on age, sex and educational level (*subsample 1*), 1232 statin users and non-users matched on cardiovascular risk (*subsample 2*), and 3609 of statin users and non-users who could be compared by quintile of propensity score for statin use (*subsample 3*).

### Statin use

Ten percent (n = 904) of the final study population used statins. Statin users and non-users were equally represented among participants with complete and incomplete (or no) cognitive data (20.7% versus 19.8% respectively). The median duration of statin use was 3.8 (interquartile range, 1.6–4.5) years. Mean (SD) daily dose was 1.7 (0.7) DDDs per day and median [IQR] cumulative dose over time was 2000 [90 to 5376] DDDs. Statin users were older and more often male than non-users (*Table 1*). Their educational level was also lower. In addition, statin user had lower performance on the cognitive tests than non-users: their mean RFFT score (SD) was lower than in non-users (58 (23) points vs. 72 (26) points; *P* <0.001), as was their percentage with high performance on the VAT (29% vs 43%; *P*<0.001). Statin users also had higher overall cardiovascular risk profile scores (FRS) and higher prevalence rates of individual cardiovascular risk factors (except for smoking) than non-users (*Table 1*).

**Table 1:** Characteristics of the total study population.

	Statin use		*p*-value
	Yes	No	
**N**	904	3191	
**Age, years, mean (SD)**	61 (10)	52 (11)	<0.001
**Male, n (%)**	593 (65)	1547 (48)	<0.001
**Educational level, n (%)**			
Primary school	136 (15)	263 (8)	<0.001
Lower secondary	357 (40)	855 (27)	
Higher secondary	217 (24)	888 (28)	
University	194 (21)	1188 (37)	
**Cardiovascular risk factors**			
History of vascular events, n (%)	215 (24)	92 (3)	<0.001
Diabetes mellitus, n (%)	174 (19)	81 (3)	<0.001
Hypertension, n (%)	807 (61)	449 (33)	<0.001
Systolic blood pressure, mmHg, mean (SD)	133 (19)	123 (17)	<0.001
Diastolic blood pressure, mmHg, mean (SD)	75 (9)	73 (9)	<0.001
Current smoking, n (%)	225 (25)	747 (24)	0.37
Body Mass Index, kg/m^2^, mean (SD)	28 (4)	26 (4)	<0.001
HDL cholesterol, mmol/L, mean (SD)	1.3 (0.4)	1.4 (0.4)	<0.001
Non-HDL cholesterol, mmol/L, mean (SD)	4.9 (1.1)	5.5 (1.0)	<0.001
Elevated albuminuria, n (%)	267 (29)	329 (10)	<0.001
Framingham Risk Score,^a^points, mean (SD)	14 (5)	10 (6)	<0.001
**RFFT score, points, mean (SD)**	58 (23)	72 (26)	<0.001
**High performance on VAT, n (%)**	261 (29)	1351 (43)	<0.001

Abbreviations: FRS, Framingham Risk Score; RFFT, Ruff Figural Fluency Test; SD, standard deviation; VAT, Visual Association Test.

a The Framingham Risk Score (FRS) for general cardiovascular risk includes age, sex, diabetes mellitus, smoking, treated and untreated blood pressure and lipid levels [28].

### RFFT score and statin use

**Figure 2:**
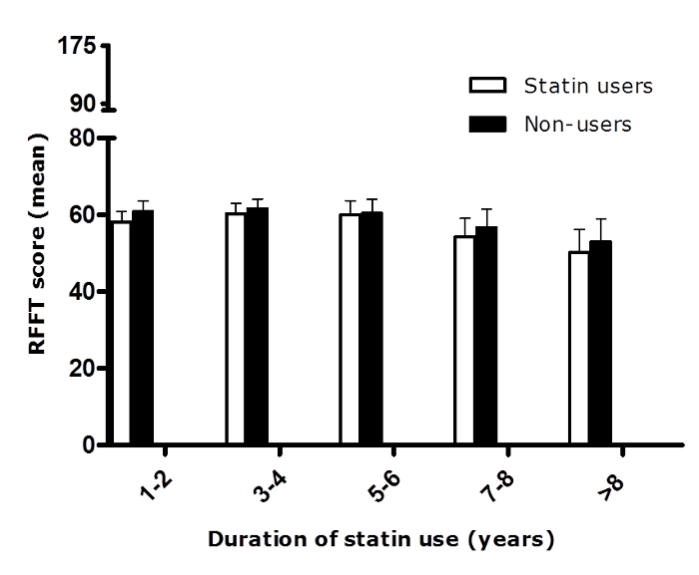
RFFT score dependent on duration of statin use. Statin users and non-users were matched on age, sex and educational level. Bars represent the upper limit of the 95% confidence intervals.

Unadjusted, statin users had worse cognitive performance than non-users. The mean RFFT score (SD) in statin users and non-users was 58 (23) and 72 (26) points, respectively (*p*<0.001). However, RFFT score was not associated with statin use (yes/no), solubility, mean dose or cumulative dose over time if subjects were matched on age, sex and educational level. RFFT score was similar among tertiles of cumulative statin dose (*Table 2*). Also, there was no association of RFFT score with duration of statin use (*Fig. 2*). For every category of duration of use, statin users had similar RFFT scores as non-users who were matched on age, sex and educational level. These findings were confirmed by multiple regression analyses. In none of the models, a statistically significant effect was found for solubility, mean dose, cumulative dose or duration of statin use (*Table 2*).

**Table 2:** Multiple linear regression analyses of the association of RFFT score with statin use.

Statin exposure	Model 1^a^			Model 2^b^		
	B	95%CI	*p*-value	B	95%CI	*p*-value
**Drug use**						
No statin	*Ref.*			*Ref.*		
Statin	−2.25	−3.86 to −0.63	0.006	−0.51	−2.28 to 1.26	0.57
**Statin type**						
Hydrophilic	*Ref.*			*Ref.*		
Lipophilic	+0.41	−2.18 to 2.99	0.76	+0.001	−2.64 to 2.64	1.0
**Cumulative dose, total DDD**						
Continuous	0.00	−0.001 to 0.00	0.095	−0.001	−0.001 to 0.001	0.98
**Categorical**						
*no statin*	*Ref.*			*Ref.*		
*Tertile 1, <1221*	−1.99	−4.50 to 0.52	0.12	+0.06	−2.56 to 2.67	0.97
*Tertile 2, 1221–2858*	−2.39	−4.90 to 0.11	0.061	−0.80	−3.39 to 1.80	0.55
*Tertile 3,>2858*	−2.34	−4.84 to 0.15	0.066	−0.77	−3.38 to 1.83	0.56
**Cumulative time exposure, years**						
Continuous	−0.07	−0.60 to 0.47	0.81	−0.14	−0.68 to 0.40	0.61
**Categorical**						
*No statin*	*Ref.*			*Ref.*		
*1–2*	−2.60	−5,14 to −0.54	0.05	−1.09	−3.70 to 1.52	0.42
*3–4*	−2.12	−4.57 to 0.18	0.07	−1.01	−3.43 to 1.41	0.42
*5–6*	−1.52	−4.98 to 1.94	0.39	−0.13	−3.65 to 3.39	0.94
*7–8*	−1.18	−5.73 to 3,36	0.61	+0.07	−4.53 to 4.67	0.98
*>8*	−4.71	−10.77 to 1.24	0.12	−3.44	−9.43 to 2.54	0.26
**Daily dose, mean DDD/day**						
Continuous	−0.98	−1.74 to 0.22	0.12	−0.19	−1.02 to 0.63	0.64
Categorical						
*no statin*	*Ref.*			*Ref.*		
*low dose, ≤1.5*	−1.82	−4.24 to 0.60	0.14	−0.28	−2.80 to 2.25	0.83
*high dose,>1.5*	−2.48	−4.38 to 0.58	0.11	−0.64	−2.68 to 1.40	0.54

Abbreviations: CI, confidence interval; DDD, defined daily dose; DM, diabetes mellitus; Ref., reference category.

a Model 1: adjusted for age, sex and educational level.

b Model 2: adjusted for age, sex, educational level, DM, hypertension, history of cardiovascular disease, smoking, BMI, albuminuria and lipid levels.

**Figure 3:**
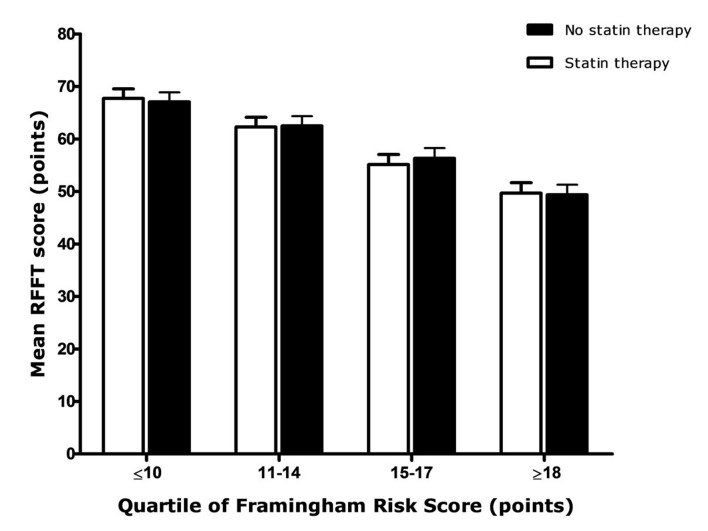
RFFT score dependent on cardiovascular risk score as measured by the Framingham Risk Score. Statin users and non-users were matched on Framingham Risk Score (FRS).**^28^** A higher FRS is associated with a higher risk of cardiovascular and cerebrovascular events within the forthcoming 10 years. RFFT scores were only adjusted for education level as age, sex and cardiovascular risk factors are included in the FRS. Bars represent the upper limit of the 95% confidence intervals.

RFFT score was dependent on overall cardiovascular risk and decreased with increasing FRS (*Fig. 3*). Mean RFFT score decreased from 67 points in persons with the lowest FRS to 49 points in persons with the highest FRS. However, in all FRS categories, statin users and non-users had comparable RFFT scores (*Fig. 3*). Similarly, RFFT score was dependent on propensity score for statin use and mean RFFT scores decreased with increasing propensity score (*Table 3*). There was however no difference between statin users and non-users within each quintile of propensity score.

**Table 3:** RFFT score in subjects with and without statin use per quintile of propensity score.

Quintile of Propensity Score^a^	Statin use	Number	Mean RFFT	95% CI	*p*-value
**1**	No	785	86.2	84.6 to 87.8	0.55
	Yes	30	83.7	76.0 to 91.4	
**2**	No	743	77.8	76.0 to 79.6	0.20
	Yes	72	73.9	68.3 to 79.5	
**3**	No	689	67.7	65.9 to 69.5	0.87
	Yes	125	67.4	63.3 to 71.5	
**4**	No	591	60.9	59.0 to 62.8	0.20
	Yes	223	58.6	55.6 to 61.6	
**5**	No	365	51.5	49.4 to 53.6	0.85
	Yes	450	51.7	50.0 to 53.4	

a The propensity score included age, sex, educational level, history of cardiovascular disease, smoking, diabetes mellitus, hypertension, BMI and albuminuria.

### VAT score and statin use

Unadjusted, statin users had worse cognitive performance than non-users. VAT performance was high in 261 (29%) statin users and 1351 (43%) non-users (p<0.001). However, VAT score was not associated with statin use (yes/no), solubility, mean dose, cumulative dose or duration of statin use over time if subjects were matched on age, sex and educational level. VAT score was similar among tertiles of cumulative statin dose (*S1 Fig* *.*). Logistic regression analysis in the overall study population yielded similar results. After correction for age, sex and educational level the odds ratio for low performance on the VAT was not significantly lower for statin users (OR −0.146, 95%CI −0.41 to 0.12, *P* = 0.284). Further adjustment did not change results. There was no significant association of the VAT score with any of the other determinants of statin use (data not shown).

VAT score was also dependent on overall cardiovascular risk and decreased with increasing FRS. In the total study population the percentage of subjects with low performance on the VAT score gradually increased from 58% in the group with the lowest FRS to 74% in the group with the highest FRS (*P for trend* <0.001). Within all FRS categories, statin users and non-users had comparable VAT scores. Similarly, there was no significant difference in VAT score between statin users and non-users within quintiles of propensity score (*data not shown*).

Elevated albuminuria was present in 14% of the study group. Various sensitivity analyses in the subsample of the PREVEND cohort which is representative for the general population (7.5%) in the different subpopulations gave essentially similar results.

## Discussion

In this large cross-sectional study, statin use was not associated with cognitive function. This was not only found in persons with low cardiovascular risk but also in persons with high cardiovascular risk, and in younger as well as older subjects. Even statin users who used high doses of statins or used statins for more than 8 years had a similar cognitive performance as non-users. Thus, it is unlikely that the lack of effect in previous RCTs was due to the relatively short treatment period [7], [8].

So far, the underlying mechanisms by which statins might affect cognitive function are not unraveled. Studies on lipid profile and cognitive function yield contradictory results. When measured in midlife, high cholesterol levels associate with an increased risk of late-life dementia and cognitive decline [4], [5]. However, late-life elevated cholesterol levels are not related to cognitive function, or inversely related [4], [5], [31]–[33]. Similarly, studies on statin use and cognitive function also showed diverting results. Several observational studies demonstrated that statin users had less cognitive decline or lower risk of developing dementia [9]–[16], while others found no differences [34]–[36]. Moreover, in some positive studies, the effect of statin use was inconsistent for different statins as well as for different outcome measures [11], [12], [16]. These contradictory results have been attributed to various limitations of the studies such as highly selected study samples (mostly aged ≥60 years), varying statin types, short treatment durations or other possible confounders [18]. Although many of these limitations were overcome in our study, we still did not find a beneficial effect of statins of cognitive function.

Despite plausible neuroprotective benefits of statins through improved cholesterol metabolism, stroke reduction and pleiotropic effects (e.g. improving endothelial function, inhibiting oxidative stress) evidence for sustained cognitive benefit is restricted. In general, neurodegenerative pathologies probably have multifactorial determinants which separately add to (the severity of) cognitive impairment [37], [38]. It could be hypothesized that among these multifactorial determinants, the effect of statins may be too small to make a difference in cognitive function.

### Strengths and Limitations

Some limitations of this study must be acknowledged. At first, the design of this study does not formally allow a conclusion on a causal relationship between variables. Second, an inherent limitation of any observational analysis includes indication bias. In our study this includes the initiation of statin treatment in people with increased cardiovascular risk. By creating subgroups of statin users and non-users with comparable cardiovascular risk profile and subgroups with comparable propensity score for statin use we aimed to minimize this bias. We do not assume any bias induced by selective drop-out of (non) statin users who refused cognitive testing, as statin users and non-users were equally represented among participants with complete and incomplete (or lacking) cognitive data. Third, the primary outcome measure was based upon a single cognitive test, mainly investigating executive functions controlled by the frontal lobe. We have to acknowledge that our cognitive tests, like other measurements of cognition (e.g. the Mini Mental State Examination (MMSE), Trail-Making Test (TMT) or Modified Telephone Interview for Cognitive Status (TICS-M) are relatively rough measurements of cognition which may not be sensitive enough to detect changes in cognition apparent to the patient. However, the RFFT is a reliable composite test which is not limited by a ceiling or floor effect and thereby more sensitive to subtle changes in cognitive performance in both young and old persons as compared to the MMSE, TMT or TICS-M [24]. Moreover, the main findings were confirmed using performance on the VAT. Fourth, the use of a computer database is an imperfect measure of adherence to statin therapy. Nevertheless, we think that pharmacy-based data are more reliable than self-reported statin therapy as used in previous studies. Finally, the PREVEND cohort is enriched for elevated albuminuria which could induce selection bias, as albuminuria is a risk factor for CVD.^20^ However, a sensitivity analysis in a subsample representative for the general population did not change results.

Our study has several strengths. Our population-based cohort included a wide age range from 35 to 82 years. Moreover, the prevalence of statin use in our population (10%) reflects the prevalence of statin use in the Dutch population [39]. Therefore, the generalizability of our data is probably well preserved. Notable other strengths of our study are that participants were well phenotyped with respect to cardiovascular risk and cognitive performance, the long follow-up of statin use (>6 years in 15% of statin users) and the detailed data on statin use obtained from a computerized pharmacy database. Previous studies used dichotomized or self-reported statin use as main determinant and had shorter durations of follow-up, although it could be argued that follow-up should be even longer than in our study as clinically relevant cognitive dysfunction might not take several years but several decades to develop. Finally, to our knowledge, we are the first to report on an in-depth analysis of statin use (including type, duration and dosage) and cognitive function in a large population-based cohort.

In conclusion, this large population-based cohort, statin use was not independently associated with better cognitive function. Statin users with long duration of use or high doses of statins had a similar cognitive performance as non-users. There was no difference in results in persons with either low or high cardiovascular risk, or in older versus younger subjects. Our findings add to the current knowledge that neither early-life nor long exposure to statins is associated with preserved cognitive function. In our opinion, there is no support for a relevant therapeutic benefit of statin use on cognitive function.

## Supporting Information

S1 Fig **Percentage of subjects with low VAT score in statin users and non-users dependent on tertile of cumulative statin dose (DDD).** Statin users and non-users were matched on age, sex and education level. Bars represent the 95% confidence interval [95% CI].
(TIF)
