# Correction: Statin Use and Cognitive Function: Population-Based Observational Study with Long-Term Follow-Up

**DOI:** 10.1371/journal.pone.0118045

**Published:** 2015-02-06

**Authors:** The PLOS ONE Staff

## Notice of Republication

This article was republished on January 2, 2015, to replace incorrect figures. The publisher apologizes for the error. Please download the article again to view the correct version.
