# Supplementary figures and images for: Statin Use and Cognitive Function: Population-Based Observational Study with Long-Term Follow-Up
Source: PLoS One. 2014 Dec 26;9(12):e115755. doi: 10.1371/journal.pone.0115755 (PMC4277319; doi:10.1371/journal.pone.0115755)

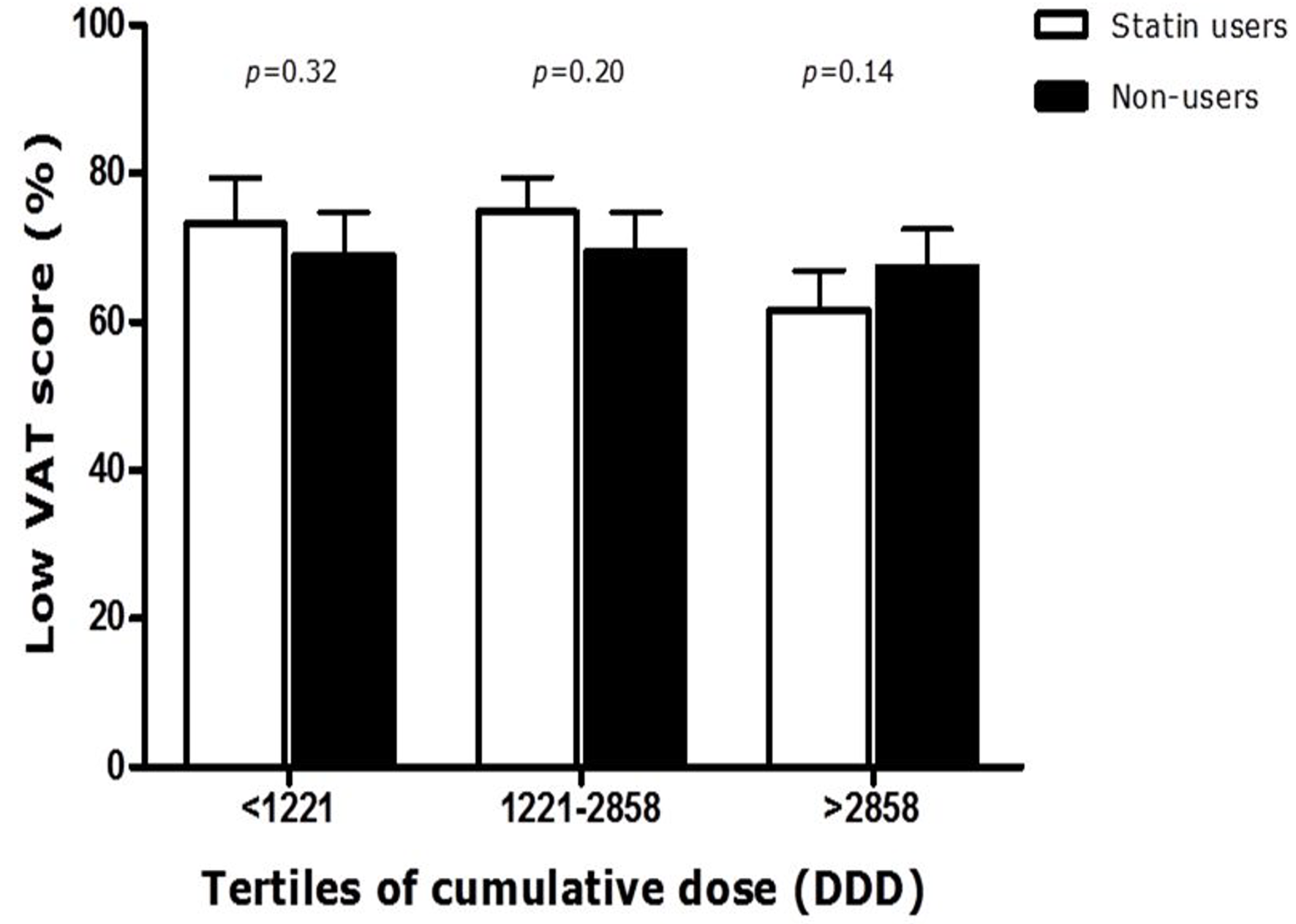

Supplement: S1 Fig — Percentage of subjects with low VAT score in statin users and non-users dependent on tertile of cumulative statin dose (DDD). Statin users and non-users were matched on age, sex and education level. Bars represent the 95% confidence interval [95% CI]. (TIF) [file pone.0115755.s001.tif]
